# Biodetoxification of Phenolic Inhibitors from Lignocellulose Pretreatment using *Kurthia huakuii* LAM0618^T^ and Subsequent Lactic Acid Fermentation

**DOI:** 10.3390/molecules23102626

**Published:** 2018-10-12

**Authors:** Yuejiao Xie, Qing Hu, Guodong Feng, Xu Jiang, Jinlong Hu, Mingxiong He, Guoquan Hu, Shumiao Zhao, Yunxiang Liang, Zhiyong Ruan, Nan Peng

**Affiliations:** 1 State Key Laboratory of Agricultural Microbiology, College of Life Science and Technology, Huazhong Agricultural University, Wuhan 430070, China; yjxie@webmail.hzau.edu.cn (Y.X.); huqing179147@163.com (Q.H.); fengguodong_hzau@hotmail.com (G.F.); shumiaozhao@mail.hzau.edu.cn (S.Z.); fa-lyx@163.com (Y.L.); 2 Key Laboratory of Microbial Resources (Ministry of Agriculture, China), Institute of Agricultural Resources and Regional Planning, CAAS, Beijing 100081, China; jiangxu@caas.cn; 3 State Key Laboratory of Agricultural Microbiology, College of Resources and Environment, Huazhong Agricultural University, Wuhan 430070, China; hujinlong-fer@hotmail.com; 4 Key Laboratory of Development and Application of Rural Renewable Energy (Ministry of Agriculture), Biomass Energy Technology Research Centre, Biogas Institute of Ministry of Agriculture, Chengdu 610041, China; hemingxiong@caas.cn (M.H.); huguoquan1@hotmail.com (G.H.)

**Keywords:** biodetoxification, phenolic inhibitors, *Kurthia huakuii* LAM0618^T^, *bacillus coagulans* LA204, simultaneous saccharification and fermentation, lactic acid

## Abstract

Phenolic inhibitors generated during alkaline pretreatment of lignocellulosic biomasses significantly hinder bacterial growth and subsequent biofuel and biochemical production. Water rinsing is an efficient method for removing these compounds. Nevertheless, this method often generates a great amount of wastewater, and leads to the loss of solid fiber particles and fermentable sugars. *Kurthia huakuii* LAM0618^T^, a recently identified microorganism, was herein shown to be able to efficiently transform phenolic compounds (syringaldehyde, hydroxybenzaldehyde, and vanillin) into less toxic acids. Taking advantage of these properties, a biodetoxification method was established by inoculating *K. huakuii* LAM0618^T^ into the NH_3_/H_2_O_2_-pretreated unwashed corn stover to degrade phenolic inhibitors and weak acids generated during the pretreatment. Subsequently, 33.47 and 17.91 g/L lactic acid was produced by *Bacillus coagulans* LA204 at 50 °C through simultaneous saccharification and fermentation (SSF) from 8% (*w*/*w*) of NH_3_/H_2_O_2_-pretreated corn stover with or without *K. huakuii* LAM0618^T^-biodetoxification, indicating biodetoxification significantly increased lactic acid titer and yield. Importantly, using 15% (*w*/*w*) of the NH_3_/H_2_O_2_-pretreated *K. huakuii* LAM0618^T^-biodetoxified corn stover as a substrate through fed-batch simultaneous saccharification and fermentation, high titer and high yield of lactic acid (84.49 g/L and 0.56 g/g corn stover, respectively, with a productivity of 0.88 g/L/h) were produced by *Bacillus coagulans* LA204. Therefore, this study reported the first study on biodetoxification of alkaline-pretreated lignocellulosic material, and this biodetoxification method could replace water rinsing for removal of phenolic inhibitors and applied in biofuel and biochemical production using the alkaline-pretreated lignocellulosic bioresources.

## 1. Introduction

Lignocellulose, the most globally abundant renewable bioresource, is attracting increasing attention in the context of biofuel and biochemical production, e.g., lactic acid, biolipids, ethanol, etc., production [1,2,3,4]. Lignocellulose mainly consists of cellulose, hemicellulose, and lignin; however, direct utilization of cellulose and hemicellulose is difficult because of their solid crystalline structure. Thus, a pretreatment step is essential to overcome this biorecalcitrance. Feasible pretreatments generally include chemical methods (dilute acid, alkaline, or alkaline/oxidative treatments), physical methods (high temperature pyrolysis, microwaving, or crushing), physicochemical methods (ammonia fiber explosion or steam explosion), and biological methods [5,6,7,8].

Chemical pretreatments are widely used to dissolve lignin, thereby improving the efficiency of enzymatic hydrolysis and subsequent fermentation [7]. Many studies have compared the advantages and disadvantages of these pretreatments, and their applicability for efficient production of a variety of biochemicals [5,6,7]. Dilute acid, dilute alkaline, and alkaline peroxide pretreatments were compared, with wheat straw and corncob as substrates. These comparisons revealed that the alkaline peroxide pretreatment is the most appropriate method for ethanol and lactic acid production even without rinsing of the pretreated substrates [8,9]. In addition, the alkaline peroxide pretreatment retained more of the hemicellulose than during other pretreatments, and dissolved a portion of the lignin, promoting enzymatic hydrolysis and reducing the inhibitory effect of lignin derivatives on subsequent fermentation [8]. 

Although chemical pretreatments are a simple and efficient way of pretreating lignocellulosic materials, these pretreatments inevitably generate several types of soluble inhibitors, such as furan derivatives (furfural and hydroxymethylfurfural [HMF]), generated during dilute acid pretreatments; and phenolic compounds as well as formate and acetate, generated by alkaline pretreatments [8,9,10]. For example, 2~5 g/L total phenolic inhibitors was detected in the lactic acid fermentation cultures using NH_3_/H_2_O_2_-pretreated corncob as substrate [9]. These compounds inhibit microbial activity and enzyme hydrolysis, which in turn hinders the industrial production of biofuels and biochemicals [11,12,13]. Various types of detoxification strategies have been investigated to mitigate the effects of inhibitors on fermentation, e.g., water rinsing, evaporation, organic solvent extraction, ion exchange adsorption, alkaline adjustment, activated carbon adsorption, oxidation, the use of lignin-blocking additives, and biodetoxification [11,12,14,15,16,17,18]. Water rinsing is the most effective method for the removal of inhibitors. However, this method results in a large amount of wastewater and loss of biomass. 

Biodetoxification refers to the use of specific enzymes (e.g., laccase and peroxidase) and microorganisms to degrade toxins or inhibitors in the lignocellulosic hydrolysates. Compared with other detoxification methods, biodetoxification has the advantage of mild reaction conditions, complete conversion of the inhibitors to non-toxic derivatives, low energy consumption, lower wastewater generation, and lower biomass loss [11,19]. A variety of microorganisms have been used for biodetoxification. The furfural-tolerant bacterium *Enterobacter cloacae* GGT036 was reported to convert 62.8% and 64.3% of furfural at their concentrations of 20 mM and 40 mM to furfuryl alcohol after a 12 h incubation, respectively [20]. A yeast strain, *Issatchenkia occidentalis* CCTCC M 206097, reduced 66.67% of syringaldehyde, 73.33% of furulic, 62% of furfural, and 85% of 5-HMF after 24 h of detoxification [21]. The oleaginous yeast *Trichosporon fermentans* converted 7 mM furfural to furfuryl alcohol after a 12 h fermentation, and then converted furfuryl alcohol to furoic acid within 240 h [22]. *Coniochaeta ligniaria* NRRL30616 was found to remove >95% of acetate, and >65% of HMF, furfural, and phenolic compounds generated during liquid hot water-pretreatment of corn stover [16]. The fungal strain *Amorphotheca resinae* ZN1 was reported to have the ability to degrade the inhibitors generated during dilute acid-pretreatment of corn stover [2,19]. Furfural/HMF were converted to furfuryl/HMF alcohols and furoic/HMF acids by *A. resinae* ZN1 under aerobic conditions, while only furfuryl/HMF alcohols were detected under anaerobic conditions [23]. Finally, *A. resinae* ZN1-detoxified and acid-pretreated corn stover was successfully used for lactic acid fermentation [24,25].

Although the use of several microorganisms for biodetoxification was reported, few microorganisms have been used for the degradation of lignin-derived inhibitors, i.e., phenolic compounds, generated during alkaline pretreatment of lignocellulosic materials. Recently, a novel bacterial strain *K. huakuii* LAM0618^T^ was isolated from biogas slurry samples [26]. It encodes a laccase capable of oxidizing typical laccase substrates, e.g., 2,6-dimethoxyphenol and l-dopamine [27]. In the current study, we confirmed that *K. huakuii* LAM0618^T^ is able to degrade phenolic compounds and formate/acetate. We further used the *K. huakuii* LAM0618^T^-biodetoxified alkaline-pretreated corn stover for high-titer lactic acid production by *Bacillus coagulans* LA204 through a simultaneous saccharification and fermentation (SSF) process. Therefore, our work represented the first study on biodetoxification of alkaline-pretreated lignocellulosic materials for biochemical production.

## 2. Results

### 2.1. K. huakuii LAM0618^T^ Degrades Phenolic Inhibitors in a Rich Medium

**Figure 1:**
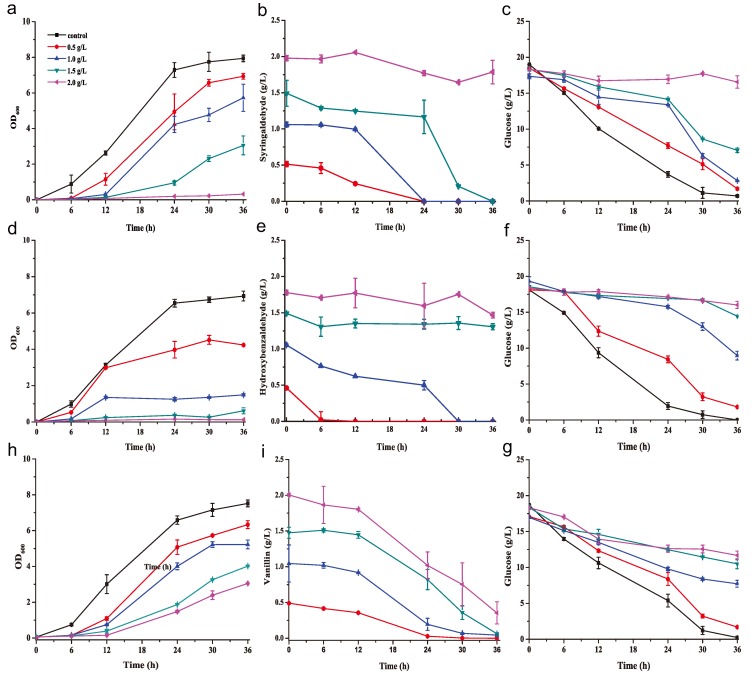
Degradation of phenolic inhibitors by *K. huakuii* LAM0618^T^ in a rich medium. Growth curve of *K. huakuii* LAM0618^T^, inhibitor degradation curve and glucose consumption curve in a rich medium containing syringaldehyde (**a**–**c**), hydroxybenzaldehyde (**d**–**f**), and vanillin (**h**–**g**) at 0.5, 1.0, 1.5, or 2.0 g/L, respectively. All experiments were performed in duplicate and results are presented as mean ± SD. Error bars indicate SD.

*K. huakuii* LAM0618^T^ was inoculated into yeast extract–peptone–dextrose (YPD) medium containing different concentrations of the phenolic inhibitors syringaldehyde, hydroxybenzaldehyde, and vanillin, which are used as the model chemicals to study the effects of phenolic inhibitors on biofuels and biochemical fermentation from lignocellulosic hydrolysates (Figure 1). Importantly, *K. huakuii* LAM0618^T^ cells showed resistance to 0.5–1.5 g/L syringaldehyde, and were sensitive to 2.0 g/L syringaldehyde (Figure 1a). Correspondingly, 0.5 and 1.0 g/L syringaldehyde was completely degraded at 24 h and 1.5 g/L syringaldehyde was completely degraded at 36 h, while 2.0 g/L syringaldehyde was not degraded (Figure 1b). Glucose was completely consumed in the control medium, while more glucose remained in the medium with higher concentration of syringaldehyde (Figure 1c). Similar results were found in the media containing hydroxybenzaldehyde, and vanillin (Figure 1d–g). Syringaldehyde, hydroxybenzaldehyde, and vanillin were completely degraded at low concentration, with degradation rates of 0.08 g/L/h for hydroxybenzaldehyde, and 0.02 g/L/h for both syringaldehyde and vanillin (Figure 1b,e,i). *K. huakuii* LAM0618^T^ was more sensitive to hydroxybenzaldehyde because ≥1.0 g/L hydroxybenzaldehyde nearly completely repressed cell growth (Figure 1d). However, even under these conditions, 1.0 g/L hydroxybenzaldehyde was also completely degraded after 30 h (Figure 1e), while the glucose was not consumed due to the inhibitory effect (Figure 1f). In contrast, *K. huakuii* LAM0618^T^ was tolerant to higher concentrations of the other two phenolic inhibitors (Figure 1a,e); syringaldehyde and vanillin at the tested higher concentrations (1.0 and 1.5 g/L) were completed degraded (Figure 1b,i). Two g/L vanillin exerted a strong inhibitory effect (Figure 1h); however, 82.5% of vanillin was degraded after 36 h under this condition (Figure 1i). Generally, rapid degradation of phenolic inhibitors began once the cell mass increased significantly (e.g., after 12 h or more in all experiments) (Figure 1). 

In summary, the growth of *K. huakuii* LAM0618^T^ and degradation curves of the phenolic inhibitors (shown in Figure 1) indicated that *K. huakuii* LAM0618^T^ tolerated low concentrations of phenolic compounds (0.5 g/L and 1 g/L), and degraded these inhibitors at different rates. With the phenolic compound concentration up to 1.5 g/L, the growth of *K. huakuii* LAM0618^T^ was hindered, and the inhibitory effect of hydroxybenzaldehyde was more obvious than syringaldehyde and vanillin. 

### 2.2. K. huakuii LAM0618^T^ Degrades Formate and Acetate in a Rich Medium

**Figure 2:**
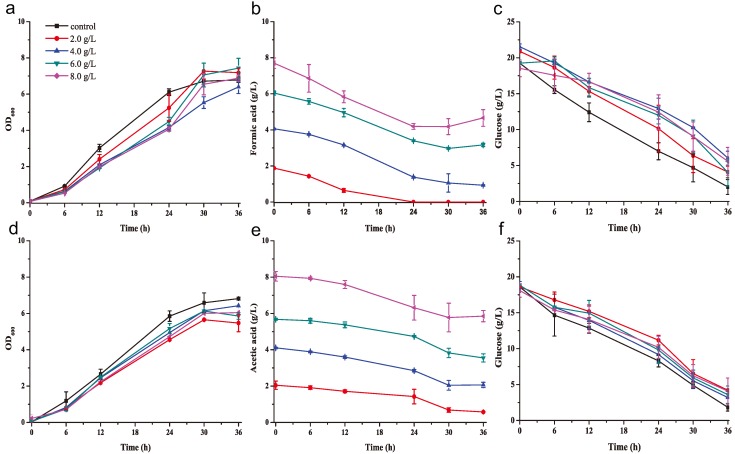
Degradation of formate/acetate by *K. huakuii* LAM0618^T^ in a rich medium. Growth curve of *K. huakuii* LAM0618^T^, formate/acetate degradation curve and glucose consumption curve in a rich medium (pH6.0) containing formate (**a**–**c**) and acetate (**d**–**f**) at 2.0, 4.0, 6.0, and 8.0 g/L, respectively. All experiments were performed in duplicate and results are presented as mean ± SD. Error bars indicate SD.

In addition to phenolic inhibitors, formate and acetate, which act as fermentation inhibitors, are also generated by alkaline pretreatment of a lignocellulosic biomass [9]. The growth of *K. huakuii* LAM0618^T^ and degradation of formate and acetate were tested in YPD medium (pH 6.0) containing different concentrations (2.0, 4.0, 6.0, and 8.0 g/L) of these inhibitors. Bacterial growth in YPD medium containing the formate or acetate was similar to that in YPD medium containing no inhibitors (Figure 2a,d), indicating that these chemicals exert only a weak inhibitory effect on cell growth. Importantly, 2 g/L formate was completely degraded within 24 h, while higher concentrations of formate were not completely degraded at 24 h and remained afterword (Figure 2b); glucose was almost consumed in the control medium and 5–8 g/L glucose remained in the medium containing formate (Figure 2c). In contrast, acetate was not effectively degraded, and 30%, 44.5%, 61.3%, and 73.6% of acetate remained in cultures with the different initial acid concentrations 36 h after the inoculation (Figure 2d); however, glucose was not consumed and 4–6 g/L glucose remained in the medium containing formate (Figure 2f). Nevertheless, *K. huakuii* LAM0618h^T^ exhibited pronounced resistance to formate and acetate even at high concentrations (Figure 2a,d). This indicated that *K. huakuii* LAM0618^T^ was able to degrade formate and acetate in an alkaline-pretreated lignocellulosic biomass.

### 2.3. K. huakuii LAM0618^T^ Trzansformed Phenolic Inhibitors into Less Toxic Acids

**Figure 3:**
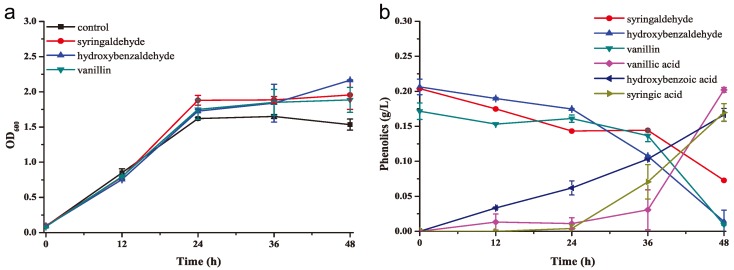
*K. huakuii* LAM0618^T^ transformed phenolic inhibitors into less toxic acids. (**a**) Growth curves of *K. huakuii* LAM0618^T^ in media containing syringaldehyde, hydroxybenzaldehyde, or vanillin (0.2 g/L) and yeast extract (3 g/L); and (**b**) degradation and their derivative acids curves of these phenolic inhibitors. All experiments were performed in duplicate and results are presented as mean ± SD. Error bars indicate SD.

Here, the ability of this strain to transform phenolic inhibitors (syringaldehyde, hydroxybenzaldehyde, and vanillin) into less toxic acids was next investigated. In this experiment, 0.2 g/L phenolic compounds were added to the medium containing 2 g/L KH_2_PO_4_, 1 g/L (NH_4_)_2_SO_4_, 1 g/L MgSO_4_, 0.5 g/L CaCl_2_, and 3 g/L yeast extract. No differences in the growth of the phenolic compounds group vs. the control were apparent during the first 24 h (Figure 3a). However, the culture optical density values at 600 nm (OD_600_) of the phenolic compounds group weakly increased after 36 h in comparison with the control group (Figure 3a); the phenolics were rapidly degraded after 36 h, accordingly (Figure 3b), indicating that they were used as the carbon sources supporting growth. We further tested the derivative acids in the medium, and found syringic acid, hydroxybenzoic acid, and vanillic acid were produced along with the degradation of above phenolic inhibitors (Figure 3b). This result revealed that the mechanism of *K. huakuii* LAM0618^T^-based bio-detoxification was transformation of phenolic inhibitors into less toxic acids.

### 2.4. Biodetoxification of NH_3_/H_2_O_2_-Pretreated Corn Stover by K. huakuii LAM0618^T^

**Figure 4:**
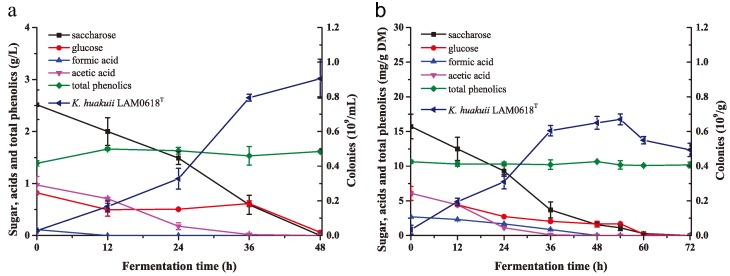
Detoxification curves by *K. huakuii* LAM0618^T^ using NH_3_/H_2_O_2_-pretreated but unwashed corn stover as substrate (**a**) through liquid cultivation with 8% (*w*/*w*) substrate loading or (**b**) through solid cultivation with 30% (*w*/*w*) substrate loading. All experiments were performed in duplicate and results are presented as mean ± SD. Error bars indicate SD.

*K. huakuii* LAM0618^T^ was used to detoxify NH_3_/H_2_O_2_-pretreated corn stover, and the efficiencies of lactic acid fermentation from *K. huakuii* LAM0618^T^-detoxified and non-detoxified corn stover were then compared. *K. huakuii* LAM0618^T^ biodetoxification was conducted in liquid and solid state. Liquid state biodetoxification was accomplished by inoculating 10% (*v*/*v*) of *K. huakuii* LAM0618^T^ into a medium containing 8% (*w*/*w*) of NH_3_/H_2_O_2_-pretreated and unwashed corn stover at 30 °C with aeration of 1.0 air volume/culture volume/min (vvm) for 2 d. Cell mass increased accompanied with decreasing saccharide and glucose (Figure 4a). During cell growth, formate and acetate were completely degraded at 12 and 36 h, respectively (Figure 4a). However, the concentration of total phenolics maintained unchanged. For solid-state biodetoxification, 10% *K. huakuii* LAM0618^T^ cell culture was inoculated into 30% (*w*/*w*) of pretreated corn stover neutralized to pH 6.5–7.0 at 30 °C for 3 d. Cell mass decreased in the initial 12 h, probably due to the high concentration of total phenolics and formate/acetate (Figure 4b). With the utilization of saccharide and glucose, cell mass increased from 12 to 36 h, and was maintained from 36 to 54 h (Figure 4b). After 60 h, saccharide and glucose were consumed, and the cell mass started to decrease (Figure 4b). Formate and acetate were degraded along with the cell growth (Figure 4b), however, the concentration of total phenolics maintained unchanged (Figure 4b), similar to that in the liquid detoxification process. 

### 2.5. Lactic Acid Fermentation from Biodetoxified and Non-Detoxified Corn Stover in SSF

**Figure 5:**
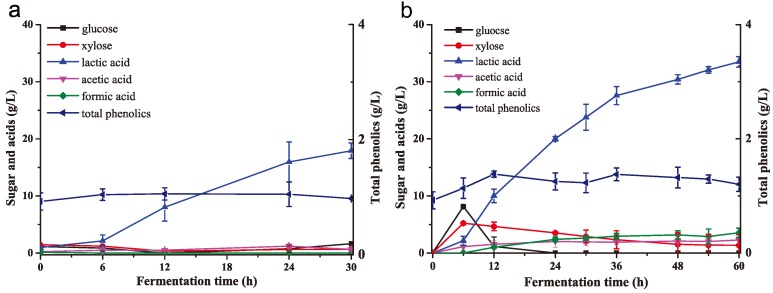
Lactic acid fermentation of biodetoxified and non-detoxified corn stover in batch SSF. *K. huakuii* LAM0618^T^-biodetoxified or non-detoxified corn stover were used as the substrates for lactic acid fermentation by *B. coagulans* LA204 in a 5 L bioreactor. Fermentation curves for glucose, xylose, lactic acid, acetic acid, and formic acid with (**a**) 8% (*w*/*w*) of NH_3_/H_2_O_2_-pretreated but unwashed corn stover, or (**b**) 8% (*w*/*w*) of NH_3_/H_2_O_2_-pretreated and biodetoxified corn stover. All experiments were performed in duplicate and results are presented as mean ± SD. Error bars indicate SD. Lactic acid titer did not grow with extension of fermentation time to 60 h in (**a**) using NH_3_/H_2_O_2_-pretreated but unwashed corn stover as the substrate.

In order to confirm the biodetoxification efficiency by *K. huakuii* LAM0618^T^, two sets of comparative SSF experiments were performed using *B. coagulans* LA204, the strain which has been previously demonstrated as a remarkably efficient producer of lactic acid [9,28]. In the non-detoxified group, 8% (*w*/*w*) of NH_3_/H_2_O_2_-pretreated and non-detoxified corn stover was used as the carbon source; 5 g/L yeast extract and 5 g/L corn steep powder were the nitrogen sources; the medium was supplied with cellulase (30 filter paper units (FPU)/g stover) and hemicellulase (30 U/g stover). During the initial stage of fermentation (6 h), lactic acid was produced slowly, with a titer of 2.13 g/L; lactic acid was produced steadily over 30 h, and the final titer reached 17.91 g/L (Figure 5a). Importantly, little residual amounts of glucose and xylose were detected during the initial stage of fermentation, suggesting that the inhibitors repressed the cellulase and hemicellulase activities (Figure 5a). The lactic acid titer and yield were 17.91 g/L and 0.22 g/g corn stover, respectively, after 30 h (Figure 5a), and they did not increase with the extension of fermentation time (data not shown). 

Biodetoxification of the NH_3_/H_2_O_2_-pretreated corn stover by *K. huakuii* LAM0618^T^ was performed at 30 °C for 2 d in a 5 L bioreactor. In the detoxification experiment, 5 g/L molasses and 5 g/L yeast extract were used as the *K. huakuii* LAM0618^T^ carbon and nitrogen sources, respectively. After biodetoxification, the nitrogen source and enzymes were added to the bioreactor, as described for the non-detoxified group. At the beginning of lactic acid fermentation, no glucose and xylose were detected (Figure 5b), indicating that molasses had been consumed by *K. huakuii* LAM0618^T^ during biodetoxification. However, liberated glucose and xylose reached 8.13 g/L and 5.22 g/L at 6 h; glucose was then rapidly consumed by *B. coagulans* LA204. The differences between the sugar curves in the non-detoxified and biodetoxified groups indicated that the cellulase and hemicellulase activities were de-repressed after biodetoxification of the NH_3_/H_2_O_2_-pretreated corn stover by *K. huakuii* LAM0618^T^. The lactic acid titer and yield reached 23.70 g/L and 0.29 g/g stover at 30 h (Figure 5b), and these values were higher than in the non-detoxified group. With the ongoing fermentation, lactic acid concentration reached 33.48 g/L, with a yield of 0.42 g/g stover after 60 h (Figure 5b). Taken together, these results indicated that the inhibitory effects of inhibitors from NH_3_/H_2_O_2_-pretreated corn stover on sugar liberation and lactic acid fermentation were relieved after the biodetoxification by *K. huakuii* LAM0618^T^. Data analyzed by ANOVA using Statistical Product and Service Solutions (SPSS) indicate that the lactic acid titer and yield were significantly higher in the medium using biodetoxified corn stover as substrate than that using non-detoxified corn stover (Table 1). 

**Figure 6:**
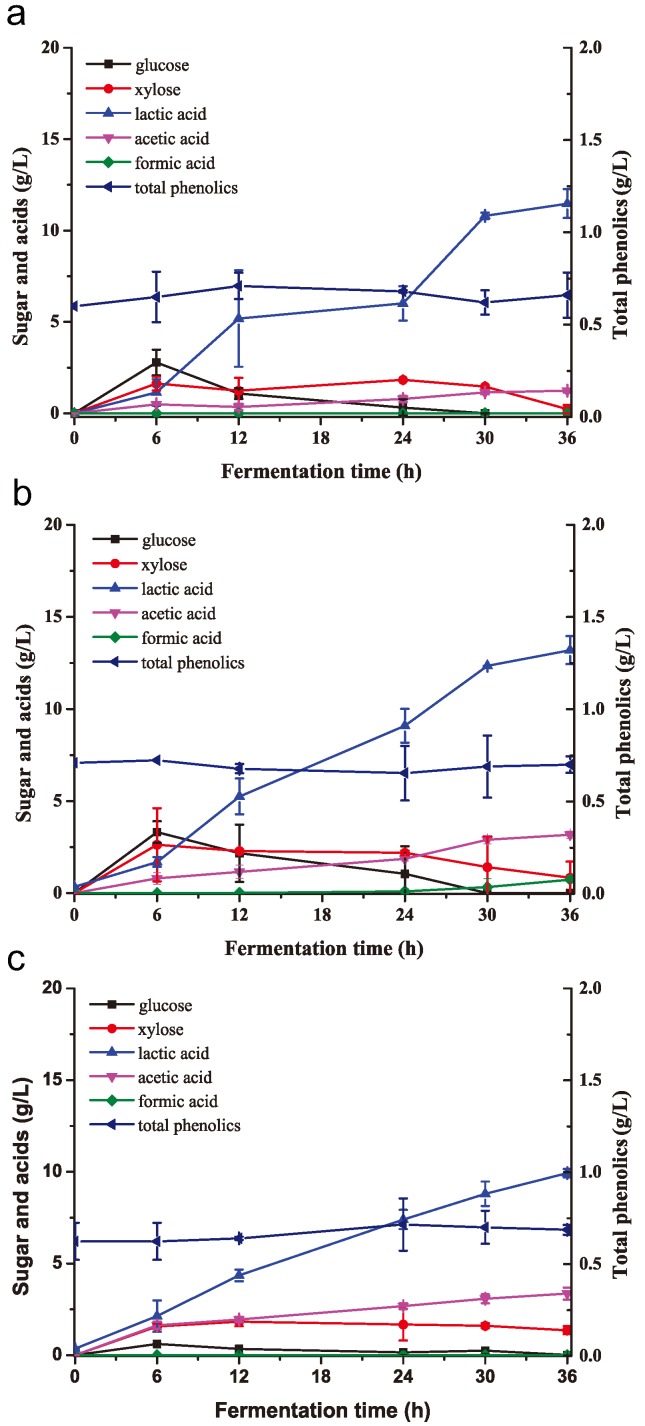
Co-culture of *K. huakuii* LAM0618^T^ and *B. coagulans* LA204 for lactic acid fermentation from 5% (*w*/*w*) NH_3_/H_2_O_2_-pretreated but unwashed corn stover in a 500 mL flask. Fermentation curves for glucose, xylose, lactic acid, acetic acid, formic acid, and total phenolic inhibitors were shown. (**a**) inoculating 10% *K. huakuii* LAM0618^T^ and 10% *B. coagulans* LA204 at 150 rpm, 30 °C or (**b**) at 50 °C, and (**c**) inoculating 10% *B. coagulans* LA204 at 150 rpm, 50 °C as the control group. All experiments were performed in duplicate and results are presented as mean ± SD. Error bars indicate SD.

However, separated biodetoxification and fermentation complicate the lactic acid production process. Here, we tested whether co-culture of *K. huakuii* LAM0618^T^ and *B. coagulans* LA204 for biodetoxification and lactic acid production could simplify this process. However, the optimal growth temperatures for *K. huakuii* LAM0618^T^ and *B. coagulans* LA204 are 30 and 50 °C, respectively. Co-culture of *K. huakuii* LAM0618^T^ and *B. coagulans* LA204 at 30 or 50 °C resulted in 11.47 or 13.19 g/L lactic acid, respectively (Figure 6a,b); however, inoculation of *B. coagulans* LA204 without *K. huakuii* LAM0618^T^ gave only 9.92 g/L lactic acid. This result indicated that biodetoxification process even at the non-optimal detoxification or fermentation conditions improved lactic acid production (Figure 6). However, the lactic acid yields were 0.23 and 0.26 g/g stover in the co-culture fermentation at 30 and 50 °C (Figure 6a,b), while the yield was 0.46 g/g stover in the separated biodetoxification and fermentation process at each optimal temperature (Table 1), suggesting co-culture was not suitable for these two strains.

### 2.6. Lactic Acid Fermentation at High-Solid Loading of Biodetoxified Corn Stover in Fed-Batch SSF

**Figure 7:**
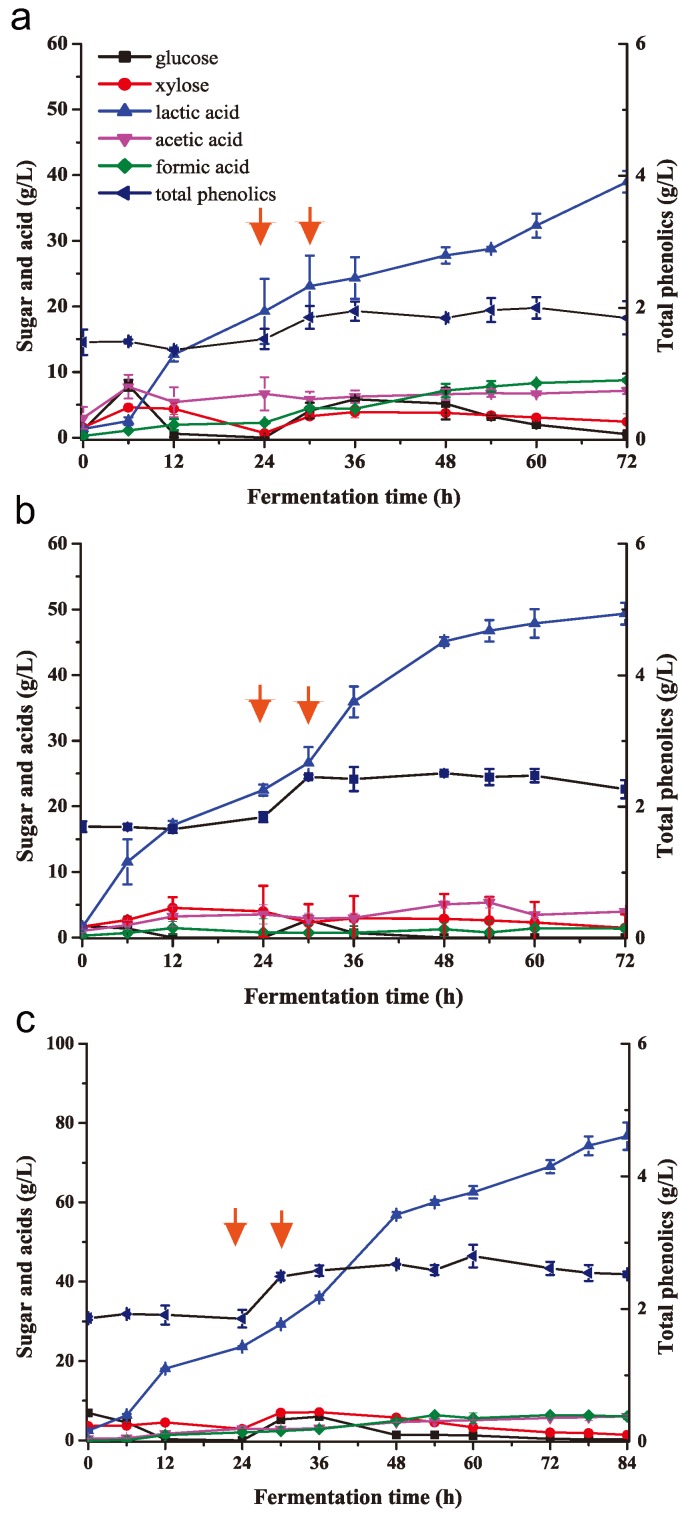
Fed-batch SSF lactic acid fermentation of biodetoxified corn stover at high-solid load using *B. coagulans* LA204. The initial 8% NH_3_/H_2_O_2_-pretreated and unwashed corn stover was biodetoxifed by *K. huakuii* LAM0618^T^ in the bioreactor. After detoxification, *B. coagulans* LA204 was inoculated for lactic acid production. Feeding was started at 24 h and ended at 30 h. (**a**) Feeding with NH_3_/H_2_O_2_-pretreated and unwashed corn stover to a final concentration of 12% (*w*/*w*); (**b**) feeding with NH_3_/H_2_O_2_ pretreated and solid biodetoxified corn stover *K. huakuii* LAM0618^T^ to a final concentration of 12% (*w*/*w*); or (**c**) feeding with NH_3_/H_2_O_2_-pretreated and solid biodetoxified corn stover *K. huakuii* LAM0618^T^ to a final concentration of 15% (*w*/*w*). Fermentation curves for glucose, xylose, lactic acid, acetic acid, and formic acid during fermentation were shown. All experiments were performed in duplicate and results are presented as mean ± SD. Error bars indicate SD. Red arrows indicate the feeding start and end time points.

Although biodetoxification of the alkaline-pretreated lignocellulosic substrate significantly improved lactic acid fermentation (Figure 5), the lactic acid titer and yield were insufficient for industrial production. It was previously reported that *A. resinae* ZN1 efficiently degraded the inhibitors generated during dilute acid-pretreatment of corn stover [2,19]. Adding the *A. resinae* ZN1–detoxified and acid-pretreated corn stover to the final concentration of 25% (*w*/*w*) resulted in high lactic acid concentration [25]. Therefore, fed-batch SSF was employed to increase the lactic acid titer. In the first fed-batch SSF experiment, 8% (*w*/*w*) of NH_3_/H_2_O_2_-pretreated and biodetoxified corn stover (2 L) was used as the initial carbon resource, and 10% (*v*/*v*) of *B. coagulans* LA204, and cellulase (30 FPU/g) and hemicellulase (30 U/g) were added at the beginning of fermentation (Figure 7a). After 24 h, 300 g of 68% (*w*/*w*) of NH_3_/H_2_O_2_—pretreated but unwashed corn stover and the enzymes were fed for 6 h, for the final corn stover concentration of 12% (*w*/*w*). The lactic acid titer reached 38.99 g/L, with the yield and productivity of 0.32 g/g corn stover and 0.54 g/L/h, respectively (Figure 7a; Table 1). The lactic acid titers at 24 h were not significantly different from the batch SSF experiment with 8% (*w*/*w*) of pretreated and biodetoxified corn stover (Figure 5b and Figure 6a; Table 1). However, inhibitors, such as phenolic compounds, were introduced into the reaction when the pretreated but non-detoxified corn stover was fed into the bioreactor. Consequently, glucose and xylose accumulated immediately after substrate feeding (Figure 7a), indicating that the inhibitors from non-detoxified corn stover hindered lactic acid fermentation. Moreover, formic acid and acetic acid also accumulated immediately after substrate feeding (Figure 7a), indicating that the inhibitors repressed cell activity preventing the degradation of weak acids. Hence, detoxified corn stover was the required feed substrate for high-solid loading fermentation. 

Nevertheless, the high concentration of corn stover in the 5 L bioreactor hindered the biodetoxification process because of high viscosity of the solution. Therefore, the NH_3_/H_2_O_2_–pretreated corn stover was detoxified using solid fermentation. In the second experiment, 8% (*w*/*w*) of NH_3_/H_2_O_2_-pretreated corn stover was detoxified by *K. huakuii* LAM0618^T^ in the 5-L bioreactor, and was then used for lactic acid fermentation as described above. Further, 278 g of 73% (*w*/*w*) corn stover detoxified in solid-state by *K. huakuii* LAM0618^T^ and the enzymes were fed from 24 h to 30 h, resulting in a final substrate concentration of 12% (*w*/*w*). Lactic acid titer reached 23.08 g/L after 24 h; the final lactic acid titer and yield were 49.35 g/L and 0.41 g/g stover at 72 h, respectively, with productivity of 0.69 g/L/h. Glucose concentration increased during substrate feeding; however, it was quickly consumed immediately after feeding (Figure 7b). Formic acid concentration was low even after substrate feeding (Figure 7b); however, after feeding the non-detoxified corn stover, the formic acid titer increased (Figure 7a). These results indicated that biodetoxification of the NH_3_/H_2_O_2_-pretreated corn stover in the course of solid fermentation reduced the inhibitor concentration and enhanced lactic acid fermentation efficiency.

Since biodetoxification in the course of solid fermentation enhanced the efficiency of lactic acid production, the third experiment was performed with the biodetoxified corn stover at 15% (*w*/*w*). The initial fermentation conditions were the same as described first experiment, with 538 g of 54% (*w*/*w*) of NH_3_/H_2_O_2_-pretreated and biodetoxified corn stover and the enzymes fed from 24 h to 30 h. At the initial stage of fermentation (0–24 h), glucose was completely consumed within the first 12 h and the lactic acid titer (23.62 g/L), yield (0.30 g/g), and productivity (0.98 g/L/h) were similar to the above two experiments. When the solid biodetoxified corn stover and the enzymes were fed into the bioreactor, glucose temporarily accumulated from 24 h to 48 h, with the lactic acid titer quickly increasing during this period. At the later stage of fermentation, accumulated xylose was utilized for lactic acid fermentation and the final lactic acid titer, yield, and productivity reached 84.49 g/L, 0.56 g/g corn stover, and 0.88 g/L/h, respectively (Figure 7c; Table 1).

Table 1 summarizes the results of lactic acid production from non-detoxified and biodetoxified corn stover. Remarkably, the lactic acid titer, yield, and productivity were significantly higher when the corn stover biodetoxified by *K. huakuii* LAM0618^T^ was used than with non-detoxified substrate under the same fermentation conditions (Table 1). This indicated that *K. huakuii* LAM0618^T^ is a good candidate microbe for the biodetoxification of lignocellulosic biomass.

## 3. Discussion

High-titer lactic acid production from alkaline- or acid-pretreated lignocellulosic materials by lactic acid bacteria has been reported recently (Table 2). However, most of the studies demonstrated that removal of inhibitors generated during alkaline- or acid-pretreatment by either water rinsing or biodetoxification is important to achieve the high titer, yield, and productivity for lactic acid fermentation (Table 2). Water rinsing is the most effective method to remove inhibitors, however, it also results in a large amount of waste water. There is a contradiction in that crop stovers, especially corn stover, are mainly produced in North China, while North China is subject to water shortage. Therefore, biodetoxification methods have been developed. However, most of these studies used acid pretreated lignocellulosic materials for biodetoxification (Table 2), and biodetoxification of alkaline-pretreated lignocellulosic materials is less reported. 

Alkaline pretreatment of lignocellulosic biomass generates phenolic inhibitors that inhibit the fermentation process [9]. *K. huakuii* LAM0618^T^ was shown to produces laccase and oxidize laccase substrates [26,27], inferring this laccase could confer detoxification activity to the cells. Here, the ability of this bacterium to degrade phenolic inhibitors was first tested. We found *K. huakuii* LAM0618^T^ efficiently degraded the phenolic inhibitors, including syringaldehyde, hydroxybenzaldehyde, and vanillin, in the rich medium (Figure 1). However, no laccase activity was detected in the culture or the cell crude extract from the detoxification experiments (data not shown). This result suggests laccase might not the main enzyme for degradation of phenolic inhibitors. 

In this study, we used *K. huakuii* LAM0618^T^ to degrade the phenolic inhibitors in the alkaline-pretreated corn stover and further used the biodetoxified stover for high-titer lactic acid fermentation (Figure 5 and Figure 6). The fermentation results showed that *B. coagulans* LA204 produced lactic acid with significantly higher efficiency from liquid- and solid-state detoxified corn stover than from non-detoxified corn stover (Table 1), indicating *K. huakuii* LAM0618^T^ is able to degrade phenolic inhibitors in the fermentation media. However, the concentration of total phenolics maintained unchanged in these media. Previously, we extended the H_2_O_2_ pretreatment time to oxidize phenolic compounds in the NH_3_-pretreated corncob. Similarly, the concentration of total phenolics was not reduced after extended oxidization, but lactic acid fermentation efficiency was enhanced using this substrate [9]. We propose that the steadiness of the total phenolic concentration is because the Folin–Ciocalteu method is used to detect total phenolic concentration. The Folin–Ciocalteu method detects the content of phenolic hydroxyl groups. However, during the detoxification, if phenolic aldehyde groups are only oxidized to the corresponding phenols, alcohol compounds, and phenolic acid compounds, phenolic hydroxyl groups on the benzene ring are still present, so the total phenolic concentration has not changed. We further detected the degradation products in the phenolic inhibitor degradation experiments, and revealed that the biodetoxification mechanism was *K. huakuii* LAM0618^T^-mediated transformation of phenolic inhibitors into less toxic acids. These results indicated the phenolic inhibitors were not completely degraded but were transformed into less toxic forms. Anyhow, the fermentation results strongly indicated that the inhibitory effect was removed when using the *K. huakuii* LAM0618^T^-detoxified corn stover as the substrate in the present study.

Lactic acid yield reached 0.56 g/g stover in this study. However, it should be noted that corn stover contained cellulose, hemicellulose, lignin derivatives, and ash after NH_3_/H_2_O_2_ pretreatment. The cellulose and hemicellulose could be converted into lactic acid, however, lignin derivatives and ash could not. We detected the composition of raw and pretreated corncob previously [9]. The cellulose and hemicellulose were 0.61–0.66 g/g pretreated corn stover and corncob on average, and the lactic acid yield was 0.56 g/g, indicating most of the usable cellulose and hemicellulose were transformed into lactic acid. This result revealed a high lactic acid yield from biodetoxified corn stover. However, a higher titer of lactic acid could inhibit fermentation because xylose was not consumed at the end of fermentation, which could lower the lactic acid yield. 

## 4. Materials and Methods 

### 4.1. Strains and Growth Conditions

Seed cultures of *B. coagulans* LA204 [28] were prepared in YPX medium (10 g/L xylose and 10 g/L yeast extract, pH 6.0), at 50 °C; seed cultures of *K. huakuii* LAM0618^T^ (ACCC 06121^T^) [26] were prepared in Luria–Bertani medium at 30 °C, with shaking at 150 rpm.

### 4.2. Raw Material and Pretreatments

Corn stover was cleaned, dried, crushed, and passed through a mesh with a circle diameter of 400 mesh (37 µm). It was pretreated using ammonium hydroxide and hydrogen peroxide (NH_3_/H_2_O_2_) as previously described [9]. Briefly, 1000 g of corn stover was pretreated with 3% NH_3_·H_2_O for 2 d, and then pretreated with 5% H_2_O_2_ for 7 d at room temperature. Following the pretreatment, the solid content of the pretreated corn stover was ca. 70% (*w*/*w*). 

### 4.3. Enzymes and Reagents

The cellulase and hemicellulase used in this study were purchased from Youtell Biochemical Co. (Yueyang, China). The cellulase activity was 119.28 ± 7.51 FPU/g; the hemicellulase activity was 31,183.50 ± 453.80 U/g. Lactic acid, formic acid, acetic acid, methanol, and acetonitrile were purchased from Sigma-Aldrich (St. Louis, MO, USA). The phenolic compounds (syringaldehyde, hydroxybenzaldehyde, and vanillin) were purchased from Titanchem Co. (Shanghai, China). 

### 4.4. Analysis of K. huakuii LAM0618^T^ Growth on Phenolic Compounds and formate/Actetae in the Presence of Glucose

Syringaldehyde, hydroxybenzaldehyde, and vanillin were added to YPD medium (20 g/L glucose, 20 g/L peptone, and 10 g/L yeast extract, pH 6.0) at 0.5, 1, 1.5, or 2 g/L; formic acid and acetic acid were added to YPD medium at 2, 4, 6, or 8 g/L and pH value was adjusted to 6.0. For the experiment, 1% (*v*/*v*) of *K. huakuii* LAM0618^T^ cell culture was incubated in 250 mL flasks containing 100 mL of YPD medium with the respective inhibitors at 30 °C, 150 rpm, for 36 h. Cell density and the inhibitor concentrations were measured every 6 h. All experiments were conducted in triplicate. 

### 4.5. Analysis of K. huakuii LAM0618^T^ Growth with Phenolic Compounds and Formate/Acetate as the Carbon Sources

Phenolic compounds (syringaldehyde, hydroxybenzaldehyde, and vanillin) and weak acids (formic acid and acetic acid) were added to 250-mL flasks containing 100 mL of a sterile medium [2 g/L KH_2_PO_4_, 1 g/L (NH_4_)_2_SO_4_, 1 g/L MgSO_4_, 0.5 g/L CaCl_2_, and 3 g/L yeast extract], at the final concentration of 0.2 g/L and 1 g/L, respectively. Then, 1% (*v*/*v*) of *K. huakuii* LAM0618^T^ cell culture was inoculated into the media and incubated at 30 °C, 150 rpm, for 36 h. Cell density and the inhibitor concentrations were measured every 12 h. All experiments were conducted in triplicate.

### 4.6. Biodetoxification of Alkaline-Pretreated Corn Stover 

*K. huakuii* LAM0618^T^ biodetoxification was conducted in liquid and solid state. Liquid state biodetoxification was accomplished by inoculating 10% (*v*/*v*) of *K. huakuii* LAM0618^T^ into a medium containing 8% (*w*/*w*) of NH_3_/H_2_O_2_-pretreated and unwashed corn stover, 5 g/L yeast extract, 5 g/L corn steep powder, and 5 g/L molasses (2 L in a 5 L bioreactor, pH 7.0), and incubating at 30 °C with aeration of 1.0 vvm for 2 d. After liquid biodetoxification, the temperature was increased to 50 °C, which was maintained for 12 h; and the medium was used for lactic acid fermentation. For solid-state biodetoxification, *K. huakuii* LAM0618^T^ cell culture was inoculated into 30% (*w*/*w*) of pretreated corn stover neutralized to pH 6.5–7.0 with 20% (*v*/*v*) of sulfuric acid; the culture contained 5 g/L yeast extract, 5 g/L corn steep powder, and 5 g/L molasses for *K. huakuii* LAM0618^T^ growth and was incubated at 30 °C for 3 d. Solid-biodetoxified corn stover was immediately used for lactic acid fermentation or stored at 4 °C.

### 4.7. Co-Culture of K. huakuii LAM0618^T^ and B. coagulans LA204 for Lactic Acid Production

The co-culture of *K. huakuii* LAM0618^T^ and *B. coagulans* LA204 experiments were carried out in 500-mL flasks containing 100 mL substrate, including 5% NH_3_–H_2_O_2_ pretreated but unwashed corn stover, 5 g/L yeast extract, 5 g/L corn steep powder, cellulase (30 FPU/g corn stover), and hemicellulase (30 U/g corn stover), then 10% *K. huakuii* LAM0618^T^ and 10% *B. coagulans* LA204 were inoculated into the flasks together and incubated at 30 or 50 °C, 150 rpm, pH 6.5. All experiments were conducted in duplicate.

### 4.8. Lactic Acid Fermentation of Biodetoxified and Non-Detoxified Corn Stover by SSF

Batch SSF was performed by inoculating 10% (*v*/*v*) of *B. coagulans* LA204 into 8% (*w*/*w*) of NH_3_/H_2_O_2_-pretreated and biodetoxified or non-biodetoxified corn stover, 5 g/L yeast extract, 5 g/L corn steep powder, cellulase (30 FPU/g corn stover), and hemicellulase (30 U/g corn stover); the initial volume was 3 L in a 5 L automatic bioreactor, and the reaction was performed at 50 °C. During lactic acid fermentation, pH was maintained at 6.0 by automatic feeding of 10 M NaOH solution. For the fed-batch experiments, the initial fermentation with 8% (*w*/*w*) of NH_3_/H_2_O_2_-pretreated and biodetoxified corn stover was set-up as described above. Then, solid-biodetoxified and non-detoxified corn stover was fed to 15% (*w*/*w*) or 12% (*w*/*w*) at 24 h. In this process, corn stover was not sterilized. Samples were collected during fermentation for sugar, acid, and inhibitor concentration determinations. All experiments were performed in duplicate under sterile conditions.

### 4.9. Analysis of Sugars, Acids, Inhibitors, and Laccase Activity

Glucose, xylose, lactic acid, acetic acid, and formic acid were analyzed using HPLC (Agilent Technologies Co. Ltd., Palo Alto, CA, USA) equipped with RID-10A detector and a Bio-Rad HPX-87H ion-exclusion column (Hercules, CA, USA). The column temperature was 40 °C; 5 mM H_2_SO_4_ was used as the mobile phase and the flow rate for sample analysis was 0.6 mL/min. Phenolic compounds and the derivatives (syringaldehyde, hydroxybenzaldehyde, vanillin, syringic acid, hydroxybenzoic acid, and vanillic acid) were analyzed using HPLC equipped with an Agilent ZORBAX Eclipse Plus C18 column (Agilent Technologies Co. Ltd., Palo Alto, CA, USA) by gradient elution, at the flow rate of 0.6 mL/min, at 30 °C, as reported previously [2]. All samples were centrifuged at 10,000× *g* for 5 min and then filtered through 0.22 µm nylon syringe filters before loading onto HPLC. The total content of phenolic compounds in the samples was determined using the Folin–Ciocalteu method [34] with gallic acid as a calibration standard. Laccase activity assay was conducted as described previously [27]. All measurements were performed in duplicate. 

### 4.10. Statistical Tests

Data were analyzed by ANOVA using Statistical Product and Service Solutions (SPSS) and results are presented as mean ± SD. Error bars indicate SD in the figures.

## Figures and Tables

**Table 1:** Summary of lactic acid production by *Bacillus coagulans* LA204 using corn stover as carbon source by SSF.

Detoxified or Non-Detoxified Corn Stover	Non-Detoxified	Detoxified	Initial: Detoxified; Fed: Non-Detoxified	Initial: Detoxified; Fed: Detoxified	Initial: Detoxified; Fed: Detoxified
Corn stover concentration (*w*/*w*)	8%	8%	8–12%	8–12%	8–15%
Fermentation time (h)	30 *^c^*	60	72	72	96
Lactic acid titer (g/L)	17.91 ± 1.11	33.47 ± 1.33	38.99 ± 1.64	49.35 ± 1.67	84.49 ± 0.95
Lactic acid yield (g/g) *^a^*	0.22	0.42	0.32	0.41	0.56
Lactic acid productivity (g/L/h) ^*b*^	0.60	0.56	0.54	0.69	0.88
Acetic acid titer (g/L)	0.65 ± 0.12	2.28 ± 0.56	7.14 ± 0.48	3.97 ± 0.17	5.39 ± 0.17
Acetic acid yield (g/g) ^*a*^	0.01	0.03	0.06	0.03	0.04
Acetic acid productivity (g/L/h) ^*b*^	0.02	0.04	0.10	0.06	0.06
α (A)	0.01		0.05		-
*p*-value	0.005		0.024		-

*^a^* g lactic acid/g corn stover; *^b^* titer of lactic acid (g/L)/fermentation time (h); *^c^* lactic acid titer did not grow with extension of fermentation time to 60 h; α (A) stands for the variance of the hypothesis; “-” stands for not tested; results are presented as mean ± SD, and error bars indicate SD.

**Table 2:** Summary of recent publications on lactic acid fermentation from detoxified and non-detoxified lignocellulosic materials through SSF.

Fermentation Strains	Substrate	Pretreatment	Fermentation Mode	Lactic Acid			Detoxification Mode	Detoxification Strains	Ref.
				Titer (g/L)	Yield (g/g)	Productivity (g/L/h)			
*Lb. pentosus* FL0421	Corn stover	NaOH	Fed-batch SSF	92.3	0.66 ^*a*^	1.92	Water rinsing		[1]
*Lb. rhamnosus and Lb. brevis*	Corn stover	NaOH	Fed-batch SSF	60.3	0.70 ^*a*^	0.58	Water rinsing		[29]
*B. coagulans* LA204	Corn stover	NaOH	Fed-batch SSF	97.59	0.68 ^*a*^	1.63	Water rinsing		[28]
*Lb. plantarum* NCIMB 8826	Corn stover	NaOH	Fed-batch SSF	61.4	0.77 ^*b*^	0.32	None		[30]
*P. acidilactici* DQ2	Corn stover	Dilute acid	SSF	101.9	0.77 ^*c*^	1.06	Bio-detoxification	*A. resinae* ZN1	[31]
*P. acidilactici* TY112	Corn stover	Dilute acid	SSF	77.66	0.65 *^c^*	1.06	Bio-detoxification	*A. resinae* ZN1	[25]
*B. coagulans* DSM2314	Sugarcane bagasse	Dilute acid	Fed-batch SSF	58.7	0.73 ^*a*^	1.81	Bio-detoxification	*B. coagulans* DSM2314	[32]
*B. coagulans* IPE22	Wheat straw	Dilute acid	SSF	46.12	0.46 ^*a*^	0.42	Bio-detoxification	*B. coagulans* IPE22	[33]
*B. coagulans* LA204	Corn stover	NH_3_/H_2_O_2_	Fed-batch SSF	84.49	0.56 ^*a*^	0.99	Bio-detoxification	*K. huakuii* LAM0618T	This study

*^a^* g/g total stover; *^b^* g/g used stover; *^c^* g/g glucose from total cellulose; -: No biodetoxification strains used. None: without detoxification.
